# Recent Advances for Drought Stress Tolerance in Maize (*Zea mays* L.): Present Status and Future Prospects

**DOI:** 10.3389/fpls.2022.872566

**Published:** 2022-05-30

**Authors:** Seema Sheoran, Yashmeet Kaur, Sushil Kumar, Shanu Shukla, Sujay Rakshit, Ramesh Kumar

**Affiliations:** Indian Council of Agricultural Research-Indian Institute of Maize Research, Ludhiana, India

**Keywords:** drought, genome editing, high-throughput phenotyping, maize, omics, QTL mapping

## Abstract

Drought stress has severely hampered maize production, affecting the livelihood and economics of millions of people worldwide. In the future, as a result of climate change, unpredictable weather events will become more frequent hence the implementation of adaptive strategies will be inevitable. Through utilizing different genetic and breeding approaches, efforts are in progress to develop the drought tolerance in maize. The recent approaches of genomics-assisted breeding, transcriptomics, proteomics, transgenics, and genome editing have fast-tracked enhancement for drought stress tolerance under laboratory and field conditions. Drought stress tolerance in maize could be considerably improved by combining omics technologies with novel breeding methods and high-throughput phenotyping (HTP). This review focuses on maize responses against drought, as well as novel breeding and system biology approaches applied to better understand drought tolerance mechanisms and the development of drought-tolerant maize cultivars. Researchers must disentangle the molecular and physiological bases of drought tolerance features in order to increase maize yield. Therefore, the integrated investments in field-based HTP, system biology, and sophisticated breeding methodologies are expected to help increase and stabilize maize production in the face of climate change.

## Introduction

The average global temperature is rising, and the environment is becoming more unpredictable, with severe drought occurring (IPCC, 2013; EPA, 2021). As per estimates, water scarcity is expected to affect 1.5–1.7 billion people in South Asia by 2050 (World Bank Group, 2021). Agriculture is the most vulnerable sector to this rapid climate change. Among many unprecedented challenges, drought is the major menace to crop production, worldwide. Drought is one of the most serious abiotic stresses, affecting human health and crop productivity in about a third of the world’s population. According to FAO estimates, drought caused a direct loss of USD 29 billion to agriculture in developing countries from 2005 to 2015 (FAO, 2021). As a result, it has become a major element in undermining the positive impacts of technological interventions, fertilization, and other contemporary developments made in recent past (Lobell et al., 2011). Hence, it is critical to develop crop varieties that, in addition to delivering higher yields, can withstand abiotic stresses like drought (Fita et al., 2015; Raza et al., 2019). Being the major biofuel and biotech (as 92% genetically modified corn in United States) crop of world, maize (*Zea mays* L.) has been anticipated to become the most important crop as its importance goes beyond food and feed (Schwietzke et al., 2009; Yadava et al., 2017). Drought is one of the major constraints in maize production as nearly 80% of wet-season maize is grown in rainfed conditions and dry season maize suffers severe water shortage (Trachsel et al., 2016). Depending on the intensity or duration of drought stress and crop stage, the maize yield losses vary from 30 to 90%, severely affecting the flowering and grain filling stages (Sah et al., 2020). In most of the countries, maize is cultivated under rainfed areas with 300–500 mm of precipitation, which is lower than the critical level to obtain decent yield (Sah et al., 2020). Since, genetic solutions are unlikely to cover more than 30% of the gap between potential and realized yield under water stress hence, the understanding of genetics and further applying it to improve drought tolerance is a key component to stabilize global maize production. The advanced molecular breeding approaches like marker-assisted selection (MAS), transgenics, and genome-editing also holds great promises to improve maize production against drought stress.

Recent studies have projected that by 2050 the global average temperature will rise and probably exceed by 2°C under the current high emission scenario (Tesfaye et al., 2018). It will cause additional maize yield loss of 10 million tons per year with increasing temperature and changing rainfall patterns (Tesfaye et al., 2018). Hence, the novel molecular approaches in genomics era have mainly targeted the dissection and manipulation of drought stress related traits, genes/quantitative trait loci (QTLs), and molecular pathways (Deikman et al., 2012; Kumar A. et al., 2022). In addition, different omics platforms also facilitate the extensive mining of the transcriptomes, proteomes and metabolomes involved in drought tolerant mechanisms (Yu D. et al., 2021). Therefore, the objective of this review is to assess the recent progress and potential benefits of novel breeding technologies made in maize to develop drought tolerance. Untangling the molecular and physiological mechanisms for drought tolerant associated traits is essential to improve crops through integrating advanced breeding tools with biotechnology.

## Morphological, Physiological, and Molecular Responses of Maize to Drought

Drought stress has a multifaceted impact on plant organization, resulting in complex and often spontaneous physiological and cellular responses. The vegetative, silking (flowering), and ear stages (grain filling) of maize are the most susceptible to drought stress, with yield losses of up to 25, 50, and 21%, respectively (Sah et al., 2020). It interferes with various molecular and physiological processes involved in plant growth and development, including photosynthetic activity, abscisic acid (ABA) accumulation, osmotic adjustment, and antioxidant capacity (Gong et al., 2014). Physiologically, when plants are stressed, they consume a large amount of adenosine triphosphate (ATP), draining their energy, and causing irreversible damage or even plant death. Plants, on the other hand, have certain adaptative mechanisms of “metabolic flexibility” that protect plants by activating apyrophosphate, which is required for transitioning poly (ADP-ribose) into ATP molecules (Hakme et al., 2008), or alternative glycolytic reactions override ATP-requiring steps, and finally the salvage pathways are induced to counteract the effects of ATP depletion (Dobrota, 2006). In response to a water deficit, adaptive traits such as osmotic adjustment, dehydration tolerance, and a reduction in photosynthetic activity emerge. Reduced photosynthetic activity results in stomatal closure and decreased photosynthetic enzyme production. Drought stress-related physiological changes at the cellular level include turgor loss and changes in membrane fluidity and composition. During drought stress, the ABA hormone concentration got increased which triggers an internal signal transduction cascade that led to reduce the osmotic potential of guard cells *via* loss of K^+^ and Cl^–^, resulting in stomatal closure (Anami et al., 2009).

Morphologically, plant growth and development are hampered due to disruptions in the cell cycle machinery (Carneiro et al., 2021). The perception of abiotic stress activates signaling cascades that stimulate cell cycle checkpoints, resulting in an impaired G1 to S transition, slowed DNA replication, and/or delayed entry into mitosis (Kadota et al., 2005; Qi and Zhang, 2020). Water stress causes meristem shortening in wheat and maize leaves and increases cell cycle duration due to decreased CDK activity (Granier et al., 2000). Changes in root and shoot growth, leading to increased root/shoot ratio, tissue water storage capacity, cuticle thickness, and water permeability are also potentially important in the long run, with root growth changes being the most important for crop plants (Verslues et al., 2006). In early days, corn plants that are severely water-stressed (WS) respond by rolling their leaves. Spikelet development is delayed, resulting in higher anthesis-silking interval (ASI), silk senescence, and pollen abortion (Lu et al., 2011; Edmeades, 2013). Stomatal closure, which reduces transpiration and CO_2_ absorption and subsequently leads to a decrease in photosynthetic activity, is one of the first reactions to drought stress (Wang et al., 2018). Severe stress during the flowering stage induces full ear abortion, and the plant becomes barren. Drought disrupts developmental stages by limiting leaf growth, plant height, and tassel architectural characteristics, which in turn reduce crop yield since plants require adequate photosynthate to acquire a necessary stature (Aslam et al., 2015). Many plants, including maize, respond to water scarcity by shifting growth and dry matter accumulation away from the shoot and toward the root (Maazou et al., 2016).

**FIGURE 1:**
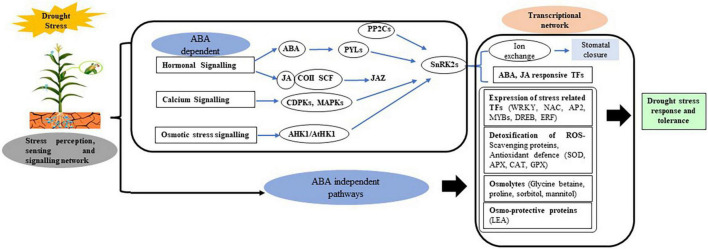
Drought stress sensing, perception, and signaling modulate transcription factors (TFs) that enhances reactive oxygen species (ROS) scavenging, protein turnover, osmotic regulation, and photosynthesis processes. ABA, abscisic acid; AP2, APETALA; Apx, ascorbate peroxidase; CAT, catalase; CDPK1, calcium-dependent protein kinase 1; DREB, dehydration responsive element binding gene; ERF, ethylene responsive factor; GPx, glutathione peroxidase; JA, jasmonic acid; LEA, late embryogenesis abundant; MAPK, mitogen-activated protein kinase; MYB, myeloblastosis oncogene; NAC, (NAM, ATAF 1/2, and CUC2) domain proteins; PP2Cs, 2C-type protein phosphatases; ROS, reactive oxygen species; SnRK2, SNF-related protein kinase 2; SOD, superoxide dismutase; TF, transcription factor; WRKY, family denoted by protein domain composed of a conserved WRKYGQK motif and a zinc-finger domain.

At metabolic level, many amino acids in the sap of WS plants increases transiently, and got accumulated only under severe water stress. Alterations in solute concentration, as well as protein–protein and protein–lipid interactions, are all metabolic changes linked to drought stress (Valliyodan and Nguyen, 2006). The synthesis of osmo-protectants and osmolytes is associated with the plant’s defense response to drought stress. As a response to water scarcity, adaptation mechanisms such as phytoalexin production, activation of the general phenylpropanoid pathway, and induction of lignin biosynthesis have evolved. Salicylic acid, methyl salicylate, jasmonic acid, methyl jasmonate, and other small molecules produced as a result of stress can also function as signaling molecules stimulating systemic defense and adaptability retorts (Nadeem et al., 2019), whereas others protect plants from oxidative damage caused by a variety of stresses, such as ascorbic acid, glutathione, tocopherols, anthocyanins, and carotenoids by scrounging the active oxygen intermediates produced. Several genes and transcription factors (TFs) have been involved and altered in order to fine-tune plant cellular metabolism in response to stress, either through osmotic homeostasis or damage control or repair, or by regulating gene expression levels. The cellular ionic, osmotic, and hormonal (ABA) signaling activates a number of stress-responsive genes which regulate gene expression patterns and provide osmotolerance and protection to plants as well as play a role in signal transduction for stress response (Lata et al., 2015; Figure 1).

## Selection Criteria for Drought Tolerance in Maize

Because drought is a multi-dimensional trait, phenotyping for drought tolerance traits is time-consuming and resource-intensive, requiring years of testing at multiple places to accurately portray the linked features (Khadka et al., 2020). Under drought stress, the direct selection for yield is often inefficient (Ziyomo and Bernardo, 2013). The practice of drought-controlled environments and deployment of secondary traits has been effective for accurate phenotyping to improve drought tolerance related traits.

The indirect selection *via* secondary traits with higher heritability and genetic correlation becomes more effective than direct selection (Ziyomo and Bernardo, 2013). The traits related to early vigor, root architecture, flowering time, carbon isotope discrimination, stomatal conductance, canopy temperature, ABA concentration, osmotic adjustment and stay green, etc., are the potential suitable secondary traits to be targeted to analyze drought stress tolerance (Kosova et al., 2014). ASI is an excellent key trait which is widely used as selection criteria in maize for drought tolerance. Generally, male and female flowering is usually synchronized within 2–3 days while under stress conditions it may be prolonged due to delay in silking and ultimately reproductive failure. Therefore, there is need to select the genotypes which have shorter ASI in case of drought stress (Araus et al., 2012). To achieve large genetic gain, the testing environment must be specifically selected. In terms of soil qualities, rainfall distribution, water distribution profiles, and possible evapotranspiration rates, the testing environment should ideally be identical to the target environment (Ribaut et al., 2009). The use of a controlled environment, as well as rigorous experimental designs and statistical analysis, will aid to increase the precision of genotypic means. To assess the G × E interactions, which is crucial for drought tolerance breeding, a multi-location evaluation is required. Recently, molecular markers related with grain yield (GY) and other traits in response to stress tolerance have been used to identify critical genes/QTLs engaged in the metabolic pathway in order to make successful selection for drought stress (Younis et al., 2020).

## Genetic Dissection of Drought Related Traits in Maize

The advancement in high-throughput genotyping and the release of reference genome dataset has made a substantial increment in conducting studies to dissect economically important traits. There are mainly two methods, i.e., linkage mapping and association mapping to widely identify complex traits associated with drought tolerance. However, detecting candidate genes remain a challenge, which has led to the development of new methods such as Mendelian randomization (MR) analysis and transcriptome-wide association studies (TWAS) to evaluate expression-trait associations and identify candidate genes linked to a trait based on gene expression variation (Zhu et al., 2016).

### Rapid Trait/Quantitative Trait Locus Mapping Through Linkage Mapping

Identification of the QTLs is an important strategy for effective selection through MAS. Since last decades, numerous studies have been undertaken to map genes or QTL associated to drought tolerance in maize (Ribaut et al., 1997; Tuberosa et al., 2002; Lu et al., 2006; Liu et al., 2011; Rahman et al., 2011; Zhu et al., 2011; Zhao et al., 2018). The advanced sequencing tools have enabled the revelation of ultra-high density genetic maps to discover massive molecular markers and precisely locate the position of QTLs. There have been multiple reports of QTLs linked to distinct phenotypes in maize under drought stress across various mapping populations (Table 1). In the series, long back Frova et al. (1999) detected QTLs for blooming time and plant height under both well-watered (WW) and WS circumstances. Nikolić et al. (2011) identified 43 QTLs for GY and morphological characteristics on all maize chromosomes except chromosome 9. They identified five QTLs for GY, which explained 0.1–15.86% of the variation. A total of 12 QTLs affecting leaf width were identified while 15 QTLs for plant height, ear height and only 1 QTL for leaf number was detected on chromosome 6. Almeida et al. (2013), identified 83 and 62 genomic regions responsible for GY and ASI, respectively, across multiple environments and diverse genetic backgrounds. Zhao et al. (2018) identified 69 QTLs for plant height, ear height, ASI, ear weight, cob weight, 100-kernel weight, and ear length under both drought-stressed and control conditions, explaining 4.0–17.2% of the phenotypic variation. Linkage mapping is a well-known method to locate QTLs but only a few QTLs are usually detected *via* this approach. Additionally, for QTL fine mapping and cloning of the genes present within it, generally large populations are required to get high map resolution (Dinka et al., 2007), making it more resource- and time-consuming process as well as the 85% of maize repetitive sequences (Schnable et al., 2009) further slows down the QTL fine mapping and cloning. The genetic dissection of drought tolerance in maize have been broadly reported but accounts of successful application of these identified QTL in maize improvement programs have been scarce. It is due to lack of validation of identified QTLs across genetic and environmental backgrounds, including genetic complexity, epistasis, profound QTL × environment interactions (QEI), population-specific QTLs, and use of agronomically poor donor lines (Vargas et al., 2006; Liu et al., 2011). Hence, meta-QTL (MQTL) analysis is adopted as another innovative tool to detect real and large effect QTL across different populations and environment. It enables the identification of genomic areas responsible for GY under both WS and WW circumstances in a variety of genotypes (Swamy et al., 2011). Zhao et al. (2018) reported 36 MQTLs in 26 populations under WW and WS conditions, as well as 39 candidate genes within those regions. Almeida et al. (2013) used MQTL analysis to find seven genomic regions for GY and one for ASI on chromosomes 1, 4, 5, 7, and 10, which were constitutively expressed in both WS and WW environments.

**TABLE 1:** Genetic dissection for drought tolerance related traits in maize.

S. No.	Parents	Population type	Marker	QTL identified	QTL identified per traits	PVE (%)	References
1.	SD34 × SD35	F_3_	RFLP	11	Grain yield (5), anthesis silking interval (3), number of ears per plants (3)	9.4–49.6	Agrama and Moussa, 1996
2.	Ac7643 × Ac7729/TZSRW	F_2_	RFLP	20	Male flowering (10), female flowering (10)	4.19–12.9	Ribaut et al., 1997
3.	Zong 3 × 87-1	RIL	SSR	17	Ear length (6), kernel number per row (5), 100-kernal weight (2), kernel weight per plant (4)	1.94–10.32	Lu et al., 2006
4.	Ac7643 × Ac7729/TZSRW	F_2:3_	RFLP	6	Anthesis silking interval (6)	1.91–7.02	Vargas et al., 2006
5.	Zong 3 × 87-1	RIL	SSR	9	Relative shoot fresh weight (5), leaf temperature difference (4)	6.2–13.1	Liu et al., 2011
6.	D5 × 7924	F_2:3_	SSR	25	Anthesis silking interval (4), grain yield (8), plant height (5), ear height (4), ear setting (4)	5.39–15.64	Zhu et al., 2011
7.	DTP79 × B73	F_2:3_	SSR	21	Sugar concentration (1), grain yield (3), leaf abscisic acid content (1), osmotic potential (4), relative water content (1), root density (1), root dry weight (1), total biomass (1), leaf surface area (8)	0.2–52.2	Rahman et al., 2011
8.	DTP79 × B73	F2	SSR	45	Grain yield (5), number of rows per ear (5), number of kernels per row (7), kernel weight (5), ear length (5), ear diameter (5), kernel length (2), Kernel width (6), kernel thickness (5)	0.1–32.08	Nikolić et al., 2013
9.	Langhuang × TS141	F_2:3_	SSR	16	Plant height (1), ear height (4), anthesis silking interval (2), ear weight (4), cob weight (3), 100-kernal weight (1), ear length (1)	4–15.77	Zhao et al., 2018
10.	Chang 7-2 × TS141	F_2:3_	SSR	17	Plant height (1), ear height (4), anthesis silking interval (1), ear weight (3), cob weight (3), 100-kernal weight (2), ear length (3)		

### Genome-Wide Association Studies for Drought Stress Tolerance in Maize

Conventional methods captured limited allelic variation for drought tolerance traits hence, genome-wide association studies (GWAS) approach has been practiced to identify favorable alleles for drought tolerance in maize. Unlike linkage mapping, it utilizes diverse populations for QTL fine mapping by harnessing the historical recombination (Schaid et al., 2018). GWAS was first applied as a candidate gene association study in maize at the beginning of the 21st century (Thornsberry et al., 2001; Beló et al., 2008). Presently, with the release of the reference genome (Schnable et al., 2009), it has been widely utilized to dissect numerous economically important traits under drought stress as mentioned in Table 2. The recent advances suggest GWAS as a prevailing tool to effectively and efficiently categorize genotype and phenotype associations. It also facilitates mapping of expression QTL (eQTL) using the transcriptomic variation, and has demonstrated the genetic basis for gene expression traits by capturing the large proportion of phenotypic variation. Fu et al. (2013), analyzed the expression of total 14,375 genes with averagely more than 15% of phenotypic variance explained by each trait per eQTL. Drought being a highly complex and influential trait makes it difficult to dissect using GWAS also. Hence, researchers use the correlated metabolic traits or seedling survival rate under drought stressed conditions and a number of association studies to detect genes involved in imparting drought tolerance in maize (Setter et al., 2011; Liu et al., 2013; Xue et al., 2013; Farfan et al., 2015; Mao et al., 2015; Zhang X. et al., 2016). In this direction, several studies have been carried to identify the loci significantly associated with tolerance related traits/genes such as accumulation of carbohydrates and ABA metabolites (Setter et al., 2011) functional dehydration responsive element binding gene (DREB) genes and other stress related genes (Liu et al., 2013; Thirunavukkarasu et al., 2014; Wallace et al., 2016) under drought. Wang et al. (2016) identified 83 genetic variants for drought resistance and located the *ZmVPP1* gene through GWAS which is demonstrated to confer drought resistance.

**TABLE 2:** Genome wide associated studies (GWAS) carried out in maize to dissect drought tolerant traits.

Population	Sample size	Traits	Markers	Marker-trait associations (MTAs) identified	References
IAP	95	Agronomic traits-7	1K	29 SNPs	Hao et al., 2011
IAP	368	Agronomic traits-14	525K	83 genetic variants, 42 candidate genes, and 1 peak SNP located on *ZmVPP1* gene	Wang et al., 2016
IAP	513	Agronomic traits-17	560K	1 candidate gene	Mao et al., 2015
IAP	318	Agronomic, metabolic, and physiological traits	157K	123 significant SNPs	Zhang X. et al., 2016
IAP	368	Seedling stage traits	525K	1 candidate gene	Liu et al., 2013
IAP	346	Agronomic traits	60K	10 QTVs	Farfan et al., 2015
IAP	240	Agronomic traits-7	30K	61 SNPs	Thirunavukkarasu et al., 2014
MAGIC	420	Seedling and germination traits	0.95K	28 significant SNPs	Rida et al., 2021
IAP	300	Grain yield and secondary traits	381K	1549 significant SNPs, 46 candidate genes	Yuan et al., 2019
IAP	224	Agronomic traits	1288K	73,573 eQTL and 97 candidate genes	Liu et al., 2020
IAP	350	Agronomic traits-9	56K	42 SNPs	Xue et al., 2013
IAP	166	Seedling traits	–	1 candidate gene	Zhang et al., 2020
IAP	209	Seedling trait–seminal root length	56K	7 candidate genes	Guo et al., 2020
IAP	309	Grain yield and related traits	58K	22 significant trait–marker associations for grain yield per plant and yield-related traits	Ma and Cao, 2021

*eQTLs, expression quantitative trait loci; IAP, inbred association panel; MAGIC, multi-parent advanced generation inter-cross; QTVs, quantitative trait variants.*

### Omics Approaches to Dissect Drought Tolerance in Maize

**FIGURE 2:**
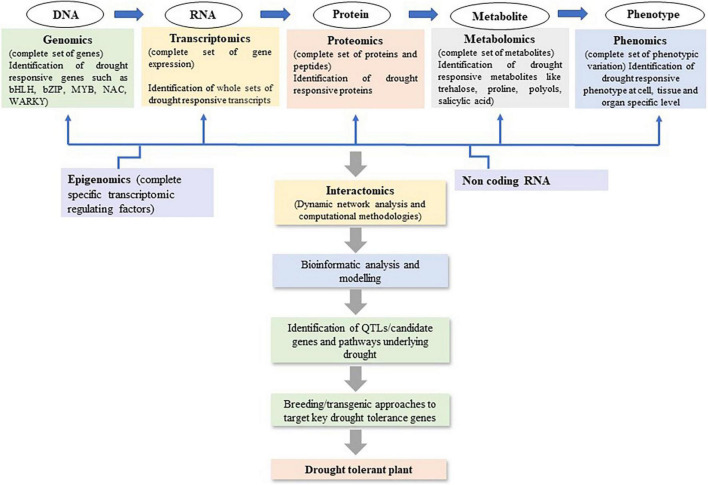
System biology-based omics approaches to strategies future basic research to decipher and develop drought tolerance maize cultivars.

Recent advancements in plant omics, including genomics, epigenomics, transcriptomics, proteomics, and metabolomics have facilitated decryption of the complex mechanisms of drought stress tolerance (Budak et al., 2015; Figure 2). As drought tolerant related traits are complex involving several regulations at gene, post-translational modifications, and protein interactions. The omics studies help to get more valuable and quality information to develop drought-tolerant crop cultivars (Wu et al., 2017). It has been used to identify the genes/QTLs, gene networks, and the regulatory pathways associated with drought stress tolerance in maize (Table 3). Genomics tools have been utilized to detect gene functions and their interactions in regulatory networks. The information related to RNA (transcriptomics), protein (proteomics), and metabolite (metabolomics) can be measured to map traits of interest onto a segregating population. Utilizing omics tools, the drought responses of maize in seeds, leaves, and roots has been extensively studied. Huang et al. (2012) investigated into maize seed desiccation tolerance and revealed differentially expressed proteins (HSP3, EMB564, and stress response protein) that could play a role in drought resistance during embryogenesis and germination. To better understand how ABA modulates the maize proteome in response to drought, Hu et al. (2011, 2012) compared the proteomic differences between the ABA-deficient maize mutant vp5 and the wild-type Vp5 under drought stress. Furthermore, several QTLs or genes associated with kernel desiccation were discovered to be involved in ABA synthesis using QTL mapping (Capelle et al., 2010) and oligo-microarray (Luo et al., 2010) analyses. Many drought tolerance related proteins or genes, such as *ZmTPA* (Huang et al., 2012), *ZmRFP1* (Xia et al., 2012), and *ZmCPK4* (Jiang et al., 2013), were also found to be induced by ABA or in an ABA-dependent manner. In the series, Bonhomme et al. (2012) also identified 3664 unique phosphorylation sites on 2496 proteins that influence epigenetic control, transcriptional regulation, cell cycle-dependent processes, phytohormone-mediated responses, cell cycle control, histone modification, DNA methylation, and ABA-, ethylene-, auxin-, or jasmonate-related responses under drought. Benešová et al. (2012) studied the drought-induced changes in the maize leaf proteome. Zhang et al. (2021) detected the role of *Bx12* and *ZmGLK44* genes in regulating metabolite biosynthesis and drought tolerance in maize while Gaffney et al. (2021) revealed several drought related biomarkers such as neophaseic acid, hydroxyabscisic acid, methyl itaconate, several phospholipids and lysolecithin, etc. The omics studies revealed that the proteins involved in maize drought response mainly include protective proteins (such as heat shock proteins, HSPs) (Hu et al., 2010; Benešová et al., 2012; Wang X. et al., 2019), stress response-related proteins (such as NBS LRR resistance-like protein) (Hu et al., 2011), 14-3-3-like proteins (Li et al., 2009; Huang et al., 2012), late embryogenesis abundant proteins (LEAs) (Huang et al., 2012; Wang X. et al., 2019), phytohormone-related proteins, and signaling proteins (such as auxin repressed protein and serine/threonine protein kinase) (Luo et al., 2010; Hu et al., 2011; Bonhomme et al., 2012; Dong et al., 2020; Li H. et al., 2021). Most of the omics studies are carried out in laboratory conditions only but there are huge differences in protein markers involved in similar metabolic pathways under field and lab conditions. Furthermore, the majority of stress treatments did not take into account the drought responses mechanism of adaptation, injury, and recovery in crops which is a major concern in such experimental designs (Lyon et al., 2016). As a result, many studies contribute only little to knowledge beyond adding some species to the list of previously discovered stress responsive proteins. Therefore, due to a lack of system biology data from field trials and huge G × E interactions in complex and unknown ways, the systematic mechanism of drought responses in crops and its application in drought tolerance development remain mainly unknown. Omics have been a valuable approach to discover the gene expressions, TFs, proteins, and pathways involved to improve drought tolerance. But the huge omics data developed for any crop drought tolerance has yet to be practically utilized at field level from laboratory. Hence, there is need to investigate the omics related data for drought responses under field conditions.

**TABLE 3:** List of studies using transcriptomics/proteomics/metabolomics approaches to unravel drought stress tolerance mechanism in maize.

Genotype	Tissue and developmental stage	Technique	Comments	References
209 maize inbred lines	Seminal roots	RNA-seq, qRT-PCR	343 differentially expressed genes (DEGs) were identified	Guo et al., 2020
Two maize RILs	Leaves	qRT-PCR	Total 613 DEGs identified at different stages in drought tolerant RIL in addition the stable expression observed of cell cycle genes, ABA-, and programmed cell death-related genes	Min et al., 2016
YE8112 (tolerant) and MO17 (sensitive)	Three-leaf-stage seedlings	RNA-seq, qRT-PCR	Upregulated LEA proteins, heat stress transcriptional factor B-2b, MYB-related TF96, pyrophosphate fructose-6-phosphate 1-phosphotransferase subunit alpha 1 (PFP ALPHA 1), chaperons, etc.	Zenda et al., 2019
287M (tolerant), 753F (sensitive)	Four-leaf stage	RNA-seq	Upregulated carotenoid cleavage dioxygenase 8, MYB-IF35, WRKY70, WRKY35, and ZFP2	Zheng et al., 2020
Zhongdi 175	Seedling stage	RNA-seq, qRT-PCR	7837 DEGs identified for drought tolerance including for sucrose metabolism and cell growth, MYB-flavonoid or MYB-bHLH-flavonoid biosynthetic pathways, ARF-Aux/IAA, bZIP/NAC-ABA, and GRAS-GA/auxin pathways	Li H. et al., 2021
Zhengdan 958	Eight-leaf stage	Multiplex iTRAQ and LC-MS/MS	Upregulated superoxide dismutase, ascorbate peroxidase, and glutathione reductase enzymes activity	Zhao et al., 2016
ND476 (tolerant), ZX978 (sensitive)	Kernel	iTRAQ, tandem mass spectrometry (MS/MS), qRT-PCR	Upregulated oxidoreductase, peroxidase and hydrolytic enzyme activities, and elevated expression of stress defense proteins	Dong et al., 2020
YE8112 (tolerant), MO17 (sensitive)	Kernel filling stage	iTRAQ Analysis, Strong Cation Exchange (SCX) and LC-MS/MS, qRT-PCR	11 up-regulated proteins under drought stress including HSPs, chaperons, late embryogenesis abundant (LEA) protein, defensin-like protein, and catalase	Wang X. et al., 2019
YE8112 (tolerant), MO17 (sensitive)	Seedling stage	iTRAQ Analysis, qRT-PCR	Total 721 differentially abundant proteins (DAPs) identified, up-regulated chlorophyll *a*–*b* binding proteins, lipid metabolism related proteins, abscisic acid stress ripening 1 (ASR1) protein	Zenda et al., 2018
B73	Leaves at seedling stage	iTRAQ, qRT-PCR, antioxidants assays	200 DAPS involved in drought signal transduction, ROS scavenging, osmotic regulation, protein synthesis, cell structure modulation, as well as other metabolisms were identified	Jiang et al., 2019
Chang 7-2 (tolerant), TS141 (sensitive)	Three-leaf stage and roots at seedling	iTRAQ with LC-MS/MS, qRT-PCR	1243 significantly differentially expressed proteins associated with associated with ribosome pathway, glycolysis/gluconeogenesis pathway, and amino sugar, and nucleotide sugar metabolism were identified	Zeng et al., 2019
10 maize hybrids	Leaves	Gas chromatography-mass spectroscopy	AA-glycine and myoinositol key metabolite for drought tolerance	Obata et al., 2015
DY 606	Leaves	^1^H NMR spectroscopy	AA-alanine, lipids-triacylglyceride and OM: malate, glutamate, formate involved as key metabolites	Sun et al., 2015
385 inbred	Leaves	LC-MS	Detected *Bx12* and *ZmGLK44* gene’s role in regulating metabolite biosynthesis and drought tolerance in maize	Zhang et al., 2021
B73	Whole plant tissues	LC-MS, high-resolution mass spectrometry	Neophaseic acid, hydroxyabscisic acid, methyl itaconate, several phospholipids, and lysolecithin detected as drought related biomarkers	Gaffney et al., 2021

*AA, amino acid; 1H-NMR, nuclear magnetic resonance; iTRAQ, isobaric tags for relative and absolute quantitation; LC-MS, liquid chromatography–mass spectrometry; OM, other metabolites; qRT-PCR, quantitative real time polymerase chain reaction.*

### Phenomics: High-Throughput and Precision Phenotyping for Drought Stress Traits

Phenomics is a new transdisciplinary area for obtaining extensive measurements of large number of plants that result in phenotypic variations over the course of their life span (Egea-Cortines and Doonan, 2018). High-throughput phenotyping (HTP) enables accelerated accurate, labor-, and cost-effective phenotyping of complex morpho-physiological traits to detect drought stress at preliminary stage (Khadka et al., 2020). According to Yang et al. (2020), advanced plant phenomics will allow for more efficient use of genetic data, leading to novel gene discovery and improved crop output and quality in the field. To make genetic progress for drought tolerance, managed stress environments (MSEs) are required, in which the severity and timing of drought stress are controlled in a way that is relevant to target environment conditions. In the absence of rain, precise water management allows stress intensity to be adjusted, maximizing the expression of genetic variability for essential secondary traits and repeating the stress pattern, which is targeted at appropriate growth phases (White et al., 2012).

The phenotypes under drought stress are dynamic and clearly defined by a series of response curves to environmental stimuli (Tardieu, 2012). The HTP systems enable the smooth running and standardization of reliable phenotypic data in glasshouse conditions by automating procedures (Li D. et al., 2021). The phenomics tools such as digital imaging, robotics and computing equipment facilitate uninterrupted measurements of thousands of plants automatically and non-destructively (Tuberosa, 2012; Fahlgren et al., 2015). For various traits such as canopy features (Fiorani et al., 2012), root architectural traits (Blouin et al., 2007), digital imaging can be used as it provides inexpensive and rapid analysis of plant features (Richardson et al., 2018). In addition, it allows to accurately analyze the root growth process at higher resolutions (Armengaud et al., 2009; Guimarães et al., 2017). Through remote-sensing *via* near-infrared spectroscopy and spectral reflectance, integrative traits should be collected with high temporal resolution (Bai et al., 2019). It helps to quantify the agronomic parameters like leaf area, yield, crop cover, biomass, etc., with evolving understanding about leaf reflectance, leaf emittance, leaf thickness, canopy shape, leaf age, nutrient status and, importantly, water status (Kumar S. et al., 2022). Dar et al. (2021) utilized HTP technique to identify drought tolerant maize lines possessing favorable traits for drought stress tolerance. Under stress, the data revealed a robust link between canopy temperature and above-ground biomass. Recent improvements in low-cost field HTPPs, high-capacity data recording, scoring, and processing, and non-invasive remote sensing approaches, together with automated environmental data gathering, aid in evaluating maize yield and other quality characteristics in drought-stricken conditions. The study of essential plant components and traits may supplement direct phenotyping in the field and advancements in user-friendly data management strategies with a more powerful interpretation of results, which have expanded the usage of field HTPPs for bumper maize production. As a result, enhancing the efficiency of crop genetic modification to fulfill the needs of future generations’ foods is based on the principles and comprehensive set of references suitable for phenotyping practices management. However, the installation of phenomics facility is highly expensive and still in infancy stage in developing countries.

### Epigenetic Modifications to Improve Drought Tolerance in Maize

Epigenetics has emerged as a novel research area to detect the biochemical changes in DNA that modulate gene expression without any change in the DNA sequence. Epigenetic changes are particularly common in response to environmental changes such as drought, and these changes can be long-lasting being heritable and through plant stress memory.

Epigenetic modifications *via* small RNA (miRNA or siRNA) for post-transcriptional gene silencing, DNA methylation, and histone changes affect gene activity and play an important role in gene expression to cope with environmental stresses (Thiebaut et al., 2019; Wojtyla et al., 2020). These modifications improve stress resistance in plants by upregulating and downregulating sRNAs (miRNAs, siRNAS) to downregulate negative regulators, upregulate positive regulators, and regulate plant hormones, reactive oxygen species (ROS), and transcriptional factors (Banerjee et al., 2017). Many advances have been made in the quantification of epigenetic variations and their impact on plant growth and development, resulting in increased yield and quality (Cortijo et al., 2014). Post-translational and post-transcriptional changes such as phosphorylation, ubiquitination, sumoylation, DNA methylation, RNA interference, histone posttranslational modifications, functional proteins, and chromatin modifications are examples of epigenetic regulation (Thiebaut et al., 2019). Using traditional breeding principles, the selection of epigenetic phenotypes can be beneficial for breeding stress-tolerant plants at various levels (metabolites, simple traits, and polygenic). Regulatory and epigenetic factors play an important role in gene expression and multi-trait development in selection process for genotype yield and quality (Verkest et al., 2015). Epigenetic changes can alter gene transcription and are an important mechanism for controlling gene expression during development in response to environmental stimulation. Basic mechanistic studies of these modifications will provide proper insight into the various genes and specific regions within them that are responsible for adapting to different abiotic stresses, resulting in a better understanding of the pathway to be targeted for crop improvement (Sun et al., 2021).

DNA methylation is a critical epigenetic modification that has been linked to plant development and stress responses (Huang et al., 2019). Various enzymes (DNA methyltransferase) that are targeted by different plant regulatory pathway systems participate in the process of catalyzing DNA methylation for a better and faster response to biotic and abiotic stresses (Zhang et al., 2018). Wang et al. (2021) generated MeDIP-Seq data to profile the DNA methylation map of four inbred lines and results showed that there were distinct drought stress responses of DNA methylation among drought-tolerant and drought-sensitive inbred lines. It was also observed that DNA methylation is involved in not only gene expression inhibition but also alternative splicing in response to drought stress. Under water stress, there was an increase in DNA methylation levels and the methylome of drought-tolerant inbred lines was much more stable than those of drought-sensitive inbred lines. Taken together, our findings may aid in deciphering the roles of DNA methylation in plant drought tolerance variations and emphasizing its role in alternative splicing.

## Breeding for Drought Stress Tolerance in Maize

### Conventional Breeding for Drought Stress Tolerance

It has been really challenging to improve drought tolerance through conventional breeding. Various drought tolerance cultivars such as Oh605, 16 tropically adapted inbred lines (TZEI 1 to TZEI 16), ND2005 and ND2006, TZE-W Pop DT STR C4, and TZE-Y Pop DT STR C4 have been successfully developed using conventional breeding methods such as recurrent selection, inbreeding, pedigree breeding, backcrossing, and hybridization (Ashraf, 2010). The success of conventional breeding depends on the germplasm diversity availability and identification of a suitable donor for tolerant genes. Hence, various germplasm sources tolerant to drought stress have been identified in maize (Cairns et al., 2013). However, the implementation of conventional breeding is highly time-consuming and cost- and labor-intensive approach. It requires repetitive number of selection and breeding cycles. In addition, it also transfers some undesirable genes causing linkage drag. The multiple genes controlling drought tolerance and their strong interactions with yield related traits make the conventional breeding methods limitedly accessible (Ahmar et al., 2020). Hence, now with progressions in next-generation sequencing, a number of advanced approaches have been implemented successfully to tailor maize for enhanced drought tolerance.

### Molecular Breeding Approaches for Drought Stress Tolerance

The application of molecular markers has improved the efficiency of breeding drought tolerant crops significantly (Andjelković and Ignjatović-Micić, 2011; Mwamahonje et al., 2021). Unlike conventional methods, genomics provides broader chances for dissecting quantitative traits into specific genetic determinants, paving the door for MAS and, eventually, cloning QTLs and manipulating them directly through genetic engineering (Tuberosa and Salvi, 2006; Oladosu et al., 2019).

Advanced genomics-assisted breeding techniques including MAS, marker-assisted back crossing (MABC), marker-assisted recurrent selection (MARS), and genomic selection (GS) have opened up new possibilities for drought tolerance enhancement. Under WS circumstances, Ribaut and Ragot (2007) employed MABC to introduce five QTLs associated to GY and flowering traits into a drought-prone maize line, observing increased grain and lower ASI. Only large effect QTLs that have been identified and validated across a wide range of genetic backgrounds are introduced into an elite variety through MABC (Bernardo, 2008). Unlike MABC, MARS uses a subset of markers that are significantly related with target traits to accumulate a high number of medium effect QTLs in a given population (Bernardo, 2008; Ribaut et al., 2010). Beyene et al. (2016) studied the genetic gains in maize GY using 10 biparental MARS populations and demonstrated its potential in increasing genetic gain under WS conditions. Another marker-based approach, i.e., GS utilizes all available significant or insignificant marker information simultaneously to envisage the genetic value of progenies for selection (Lorenz, 2013). Using GS, Beyene et al. (2015) assessed genetic gains in GY from eight bi-parental maize populations under managed drought stress environments. The study showed that GS is a more effective method for enhancing genetic gains under drought stress in tropical maize than pedigree-based traditional phenotypic selection. Through “breeding by design” strategy, all identified QTLs and trans genes relevant to yield and drought tolerance traits can be accumulated into elite genotypes. Breeders would be able to strategize new ideotype crops by analyzing allelic variations and the genetic basis of secondary traits involved in regulating drought tolerance.

The cloning of genes associated to drought tolerant QTLs is another important application of molecular breeding. By mapping of known stress responsive genes, different techniques are being employed to clone candidate genes (Salvi and Tuberosa, 2005; Tondelli et al., 2006). Masle et al. (2005), for example, cloned the ERECTA gene in *Arabidopsis thaliana*, which is a DNA sequence that is beyond a QTL for transpiration efficiency. However, there is no report in the literature on the cloning of genes underlying QTLs in any crop species, or determining a suitable QTL for gene cloning is still difficult (Hu et al., 2021). By compiling all of the aforementioned data, it is clear that molecular breeding research has not progressed beyond the detection of a specific trait under drought stressed conditions. However, to evaluate whether QTLs found in a mapping population can improve drought tolerance in high yielding elite genotypes after introduction remains a difficult task for researchers. Although the application of MAS appears to be more promising and meaningful but its contribution to the development of drought-tolerant cultivars has been modest so far with only a few successful studies available (Zhao et al., 2008; Beyene et al., 2016).

### Transgenic Approaches to Develop Drought Tolerance in Maize

The rapid advancement in biotechnology with genome sequencing has resulted in a diverse set of techniques for manipulating and identifying genes of interest involved in certain processes, such as drought responses. The current research on engineering drought-tolerant plants is primarily based on the transferring of one or more genes involved in signaling and regulatory pathways, or that encode enzymes involved in pathways leading to the synthesis of functional and structural protectants like osmolytes and antioxidants or stress tolerance conferring proteins (Vinocur and Altman, 2005; Khan et al., 2019). Genetic engineering facilitates wider range of solutions to improve drought tolerance more rapidly with reduced time (Martignago et al., 2020). Through genetic engineering techniques, a number of natural and synthetic genes and TFs have been incorporated into maize to enhance drought tolerance (Parmar et al., 2017; Table 4). A large number of drought responsive genes and TFs have been identified such as *TsVP* and *BetA* from *Thellungiella halophila* and *Escherichia coli*, *ZmPLC1*, *ZmVPP1*, *ZmTIP1*, and *ZmNAC111* from maize (Wang et al., 2008, 2016; Mao et al., 2015; Zhang et al., 2020), *SbER1–1* and *SbER2–1* from sorghum (Li et al., 2019), *LOS5* from *Arabidopsis* (Lu et al., 2013), *TsCBF1* from *T. halophila* (Zhang et al., 2010), and many more, whose overexpression result in drought tolerance development in maize *via* transgenic approach (Jia et al., 2020; Muppala et al., 2021; Yu H. et al., 2021). Generally, the DREB proteins, i.e., a subfamily of APETALA 2/ethylene-responsive element binding factor (AP2/ERF) family and mitogen activated proteins are targeted for maize drought tolerance (Bidhan et al., 2011). Shou et al. (2004) incorporated Nicotiana Protease Kinase (NPK1) *via* genetic transformation to develop transgenic maize which induces HSPs and glutathione-*S*-transferases (GSTs) to protect photosynthetic machinery during drought stress conditions. Quan et al. (2004) transformed an elite maize inbred line DH4866 with a key *E. coli* enzyme encoding choline dehydrogenase, which has been linked to maize abiotic stress. At the germination and young seedling stage, transgenic maize plants accumulated more glycine betaine and exhibited improved GY and drought resistance than non-transgenic plants. Amara et al. (2013) improved drought tolerance in transgenic maize plants by over-expressing the group LEA Rab28 candidate gene, which leads to increased Rab28 protein accumulation and stability and improved water stress tolerance. He et al. (2018) reported that the overexpression of *ZmPYL3*, *ZmPYL9*, *ZmPYL10*, and *ZmPYL13* genes played important role to impart drought resistance in transgenic plants by enhancing ABA signaling, proline, and other drought related marker genes. A transgenic gene-silencing approach was used to modulate the ethylene biosynthesis in maize and determine its effect on GY under drought stress. The results showed that there was significantly higher GY, decreased and an increase in kernel number/ear in transgene-positive events compared to nulls (Habben et al., 2014). Similarly, Liu et al. (2015) cloned maize *ZmSDD1* gene and dissected its functions and performance to drought stress. The over-expression of *ZmSDD1* in transformed maize lines converts these lines in drought-resistant lines. The expression level of *ZmSDD1* in transgenic plants was 6.68 times higher than that in wild types with reduction of stomatal density and 80% higher survival rate than that of wild type. In this direction, Monsanto company in alliance with BASF, developed the first biotechnology-derived drought tolerant maize variety, i.e., MON 87460 expressing bacterial cold shock protein B (CspB) and obtained 6% increased GY under drought conditions (Nemali et al., 2015). In maize ZmNF-YB2, the homolog of AtNF-YB, was found to be constitutively expressed in transgenic plants and outperformed *Arabidopsis* NF-YBs in terms of survival. Transgenic plants were also drought resistant in field trials, due to a number of higher stomatal conductance, cooler leaf temperatures, higher chlorophyll content, and delayed onset of senescence (Nelson et al., 2007). Recently, Muppala et al. (2021) has introduced *rpk and nced* genes into elite maize inbred lines from LeaP and SalT promoters to develop stable transgenic lines. The results showed more accumulation of ABA in transgenic plants and significant survival capacity over the control plants under stress. The improvement of drought stress tolerance is a multi-tool approach by integrating breeding, biotechnological and agronomic practices. Hence, in an attempt to develop drought tolerant maize cultivars, the research conducted utilizing transgenics declares it as one of the potential tools to manage drought stress associated risks.

**TABLE 4:** Application of transgenic and genome editing tools to improve drought tolerance in maize.

Targeted genes	Source organism	Gene expression	Traits	Status	References
**Transgenics**					
*CspA*, *CspB*	*Escherichia coli*	Overexpression	High chlorophyll content, improved photosynthetic rate, and reduced leaf area during vegetative growth	Best performing lines commercialized as Genuity DroughtGard	Nemali et al., 2015
*NF-YB1*, *NF-YB2*	*Arabidopsis*, maize	Overexpression	Higher stomatal conductance and chlorophyll content, and delayed senescence	50% yield increment under severe drought conditions. These lines not assessed in the field and never introduced to the market	Nelson et al., 2007
TPS/TPP (T6P phosphatase, TPP)	Rice	Overexpression	Altered carbon allocation and improved yield in both well-watered and water-limited field trials	Drought tolerance effects assessed in extensive field trials with yield improvement under severe drought	Nuccio et al., 2015
*TsVP*	*Thellungiella halophila*	Constitutive expression	Increased total soluble sugars and proline under osmotic stress. Improvements in dehydration tolerance	In a small-scale field experiment, these transgenic plants showed a higher yield under drought conditions than the control plants	Li et al., 2008
*Zm-ARGOS1 a*, *Zm-ARGOS8*	Maize	Overexpression	Improve dehydration avoidance in both plant species by reducing ethylene sensitivity	Reduced ethylene sensitivity and enhanced maize yields under both drought stress and well-watered conditions	Shi et al., 2015
*nced* and *rpk*	Maize	Overexpression	Play key roles in the abscisic acid pathway and upstream component in ABA signaling	Introducing two genes involved in the ABA pathway and developed stable transgenic plants with desired characters	Muppala et al., 2021
*sod*, *apx*, *gr*, *dhar*, and *mdhar*	–	Overexpression	Play key roles in the ascorbate–glutathione pathway	Fertile putative transgenic maize plants produced	Sreenu et al., 2016
**Genome editing**					
*ARGOS8*	*Arabidopsis*	Constitutive expression CRISPR/Cas9	Improved drought tolerance in a field trial under stress conditions without affecting yield in well-watered control experiments	Commercialization of these lines is under evaluation	Shi et al., 2017
*ahb2*	Maize	Gene knockout	Quicker stomatal closure in response to dehydration stress	Three independent homozygous lines (i1, d2, and d35) tolerant to drought stress obtained	Liu et al., 2020

### Genome Editing: Recent Progress to Improve Drought Tolerance in Maize

Recently, the genome editing technologies have emerged that enable rapid and precise manipulation of DNA sequences by editing major genes for development of drought-tolerant germplasm. The genome editing tools such as mega nucleases, zinc-finger nucleases (ZFN), transcription activator-like effector nucleases (TALEN), and the clustered regularly interspaced short palindromic repeat (CRISPR)/CRISPR-associated nuclease protein 9 (Cas9) system, have provided targeted gene modification in plants (Li et al., 2013; Čermák et al., 2015; Khalil, 2020). The CRISPR-Cas9 system has revolutionized both basic and applied research of plants and animals as a powerful genome editing tool (Shan et al., 2013; Wang T. et al., 2019). The approach of genome editing has been widely adopted in many plant species, such as *Arabidopsis* (Fauser et al., 2014), rice (Hu et al., 2016), wheat (Zhang Y. et al., 2016), potato (Andersson et al., 2018), tomato, and many fruit crops (Wang T. et al., 2019) to improve various traits like yield improvement, biotic and abiotic stress management. CRISPR/Cas9 based genome editing has been applied to increase crop disease resistance and also to improve tolerance to major abiotic stresses like drought and salinity (Wang T. et al., 2019). It helps to identify the important role/function of genes under different biotic and abiotic stresses through creating the particular gene knock-out or knock-down. In maize, recently a few studies have been carried out to improve drought tolerance by utilizing CRISPR-Cas technology (Table 4). Shi et al. (2017) employed a CRISPR-Cas aided advanced breeding technology to generate novel variants of maize *ARGOS8* which is a negative regulator of ethylene responses. The results showed that in comparison to wild types, the *ARGOS8* variants increased GY under drought stressed conditions. It demonstrated that modifying the expression patterns of a single native gene responsible for drought tolerance under stressed conditions can maintain yield potential. Plants derived from this method could be classified as non-GM crops. To engineer drought tolerance, multiple genes associated with complex metabolic pathways needs to be manipulated. As a result, the practical realization of drought tolerance is dependent on the use of molecular techniques capable of manipulating multiple genes at the same time. Recently, using Golden Gate cloning or the Gibson assembly method, multiple sgRNAs driven by independent promoters have been multiplexed into a single CRISPR/Cas9 expression vector (Silva and Patron, 2017). In combination with haploid induction, genome editing will be another important development in improving the breeding speed of stress tolerant maize variants in the future. In this method, the HI-Edit (haploid induction editing technology) or IMGE (haploid-inducer mediated genome editing), i.e., the haploid inducer line is also the editor line. Following the HI inducer cross, the desired genome edit occurs in the zygote, which is followed by a uniparental genome elimination (haploidization) (Malenica et al., 2021). This strategy generates edits to elite breeding lines faster, avoiding selfing or backcrossing if necessary to eliminate the transgene encoding the editing machinery. CRISPR-edited crops, on the other hand, confront socio-political obstacles, such as public acceptability and government regulation (Scheben and Edwards, 2017).

## Progress to Develop Drought Tolerant Maize Cultivars: Mega Projects

There are a number of international and national level initiatives undertaken to develop drought tolerant maize cultivars such as drought tolerant maize for Africa (DTMA), water efficient maize for Africa (WEMA), and affordable, accessible Asian (AAA) drought tolerance maize project. Under DTMA project nearly 160 drought tolerant maize varieties superior to the commercial maize varieties have been released between 2007 and 2013. These cultivars have been tested in experimental trails at farmers’ fields, and disseminated among farmers in 13 African countries through different national agricultural research systems and private seed companies (Fisher et al., 2015). As per reports, more than 2 million farmers in sub-Saharan Africa are growing these new varieties and reporting 20–30% higher yield than traditional varieties, even under moderate drought conditions. It benefited 30–40 million people and gained $160–200 million worth by increased grain production each year in drought-affected areas of sub-Saharan Africa (CIMMYT, 2021).

Another project, WEMA was in pipeline from 2008 to 2018 to develop drought-tolerant maize for smallholder farmers in Asia. Similarly, a project is ongoing at Indian Council of Agricultural Research (ICAR)-Indian Institute of Maize Research (IIMR), India for genetic enhancement of maize for the development of high yielding and climate resilient hybrids with the objective to develop drought tolerance new productive inbreds and hybrids (IIMR, 2019). Various seed companies have also released drought tolerant hybrids utilizing both traditional and transgenic breeding technologies such as Pioneer Optimum AQUAmax™, Syngenta Artesian™, and DroughtGard™ (Adee et al., 2016). Since, last few decades considering the severity of drought stress, a number of drought tolerant maize varieties have been developed under such projects throughout the globe (Table 5).

**TABLE 5:** Drought tolerant maize cultivars/genotypes developed utilizing conventional and molecular breeding tools.

S. No.	Germplasm/variety for drought tolerance	Strategy/method	Traits targeted	References
1.	CML 562, CML 563, CML 564, CML 565, CML 566, CML 567	Inbred evaluation and doubled haploidy	Drought tolerance traits	CIMMYT, 2015
3.	GDRM-187	Participatory plant breeding	Extra-early maturity	Witcombe et al., 2003
4.	ZM 309, 401, 423, 521, 623, 625, 721, KDVI 1, 4, 6, WS 103, Melkassa 4, WH 403, 502, 504, and ZMS 402, 737	Conventional breeding	–	Dar et al., 2016
6.	KSC720, KSC 710GT, and KSC 700	Field screening	Grain yield and drought tolerant indices	Naghavi et al., 2013
7.	TZEE Y POP STR QPM C0 and EVDT W 99 STR QPM CO	Mother and baby trials at field	Yield related traits	Buah et al., 2013
9.	M1227-17, M0826-3, and M1124-18	Field screening	Grain yield, ASI	Meseka et al., 2018
11.	La Posta Sequia, Pool 26 Sequia, Pool 18 Sequia, Pool 16 Sequia, DTPW, DTPY, TuxpenoSequia	FS/S1/S2 breeding schemes breeding schemes	–	Edmeades et al., 1997
26.	Malawi hybrids 30, 31, and 32	Conventional breeding	High grain yield and flint grains	CIMMYT, 2013
14.	AMDROUT1(DT-Tester) c1F2, AMDROUT2(Ac)c1F2, AMDROUT3, AMDROUT4, AMDROUT (5 × 6), MARS7 to MARS12, G16BNSEQ-C3, DTPY-C9	Genomic selection	Genetic gains per year	Vivek et al., 2017
15.	70 May 80, Aaccel, and Indaco	Drought indices	Grain yield	Barutcular et al., 2016
16.	ADL47 × EXL15, ADL41 × EXL15, and EXL02 × ADL47	Evaluation under drought stress	Grain yield	Adebayo and Menkir, 2014
17.	9011-30, STR-EV-IWD, and IYFD-C0	Evaluation under drought stress	Ears/plant and kernels/ear	Kamara et al., 2003
18.	SAMMAZ-26 (DTSTR WC1)	Improvement breeding	High grain yield	NSPI, 2022
	TL 98, 99, and 01	MABC	ASI, flowering traits, yield	Ribaut and Ragot, 2007
19.	Hybrid TA5084	Gene introgression	Yield	Syngenta Foundation, 2021
20.	MON87460	Transgenic	Yield	Nemali et al., 2015
21.	2004 TZE-WDT STR C4, 2013 DTE STR-Y Syn, DT-Y STR Synthetic, 2009 TZE-WDT STR, 2008 DTMA-Y STR, 2012 TZE-WDT C4 STR C5, 2014 TZE-WDT STR, 2011 TZE-Y DT STR	Genetic improvement breeding	Ear height, plant height, kernel row number, ear weight, grain yield	Oluwaranti and Ajani, 2016
25.	Longe 1, Longe 4, Longe 5, MM3, Longe 7H, Longe 9H, Longe 10H, Longe 11H, UH5051, UH5052, UH5053, PAN 67, WE 2101, WE 2103, WE 2104, WE 2106, WE 2114, WE 2115	Modern conventional methods	Early maturity, yield	Simtowe et al., 2019

## Conclusion and Future Perspectives

Currently, the agriculture sector is facing the biggest challenge of climatic change with frequent spells of drought. In addition, drought being a complex trait further complicates the speedily development of drought tolerant maize cultivars. A number of advanced QTL mapping, genomic-assisted breeding methods, genetic engineering, and genome editing tools have been implemented successfully to harness the commercial stress-tolerant maize varieties. With the deployment of recent advanced technologies, researchers have provided some insight on the genetic architecture and regulatory pathways of drought tolerance mechanism in maize. The rapid trait mining has detected several genomic regions regulating drought-associated traits that can be utilized for direct selection. But still the candidate genes involved to create genetic variants for these traits are unexplored. Hence, it is highly important to execute the multi-omics studies, genetic designs, and pertinent analytical methods in integration to clearly understand the molecular regulatory mechanisms of drought response. The other approaches *viz*., transgenics and genome editing have widened the range to improve desired traits in shortest period of time. Among crop plants, *Arabidopsis*, *Brachypodium distachyon*, and rice are the excellent model plants to study the drought stress responses. It provides a useful heterologous system to test new genes discovered in cereal crops like maize and their relative molecular responses. As a wild grass, *Brachypodium* has never been subjected to human selection, which is why it has a very promising framework for tracking drought-tolerance mechanisms in grasses that may have been lost during temperate cereal crop’s domestication. Hence, the research work done in these model plants should be transferred to maize by using an accurate experimental design, including high throughput phenotyping platforms to thoroughly investigate tissue- and cell type-specific drought responses. It is crucial to strengthen the coordination among different research groups and institutions working to develop drought tolerance in maize. In addition, partnership among public and private sector could not only prove instrumental to provide promising results, but also help in designing potential drought-tolerant strategies that might have a huge impact on global maize production.

## Author Contributions

SeS and YK wrote the manuscript. SK and ShS contributed in structuring the manuscript. RK conceptualized the idea. RK and SR edited the manuscript thoroughly. All authors read and finalized the manuscript.

## Conflict of Interest

The authors declare that the research was conducted in the absence of any commercial or financial relationships that could be construed as a potential conflict of interest.

## Publisher’s Note

All claims expressed in this article are solely those of the authors and do not necessarily represent those of their affiliated organizations, or those of the publisher, the editors and the reviewers. Any product that may be evaluated in this article, or claim that may be made by its manufacturer, is not guaranteed or endorsed by the publisher.
